# Clinical Application of Perioperative Nursing in Vascular Interventional Therapy

**DOI:** 10.12669/pjms.41.9.12019

**Published:** 2025-09

**Authors:** Jing Zhao, Dan Chen, Xiaojing Ren

**Affiliations:** 1 Jing Zhao Department of Anesthesiology, The First Medical Center of Chinese PLA General Hospital, Beijing 100039, P. R. China; 2 Dan Chen Department of Interventional Ultrasound, Oncology Department, The Fifth Medical Center of Chinese PLA General Hospital, Beijing 100039, P. R. China; 3 Xiaojing Ren Department of Radiology Diagnosis, The First Medical Center of Chinese PLA General Hospital, Beijing 100039, P. R. China

**Keywords:** Comprehensive nursing, Complications, Intervention, Vascular surgery, Nursing satisfaction

## Abstract

**Objective:**

To evaluate the influence and clinical outcomes of comprehensive nursing interventions on patients receiving interventional treatment for vascular surgery.

**Methodology:**

A retrospective analysis was performed on the clinical data of 80 patients who received interventional treatment in the vascular surgery department of The First Medical Center of Chinese PLA General Hospital from June 2022 to June 2024. The patients were categorized into the control group received routine perioperative nursing and the observation group provided with comprehensive nursing interventions in conjunction with the routine nursing based on the control group according to the nursing methodologies employed, each group consisting of 40 patients. Evaluate the occurrence of complications in both groups was noted and analyze the variations in comfort level, quality of life following the intervention.

**Results:**

The incidence of complications in the observation group was 10%, lower than the 30% incidence in the control group (c^2^= 4.762, P=0.029). After the intervention, the GCQ score of the observation group was higher than that of the control group and the comfort level was significantly better than that of the control group(p<0.05); the HPLP-II score of the observation group was higher than that of the control group and the quality of life was better than that of the control group (p< 0.05); the SAS and SDS scores of patients in the observation group were both lower than those of the control group (p<0.05).

**Conclusion:**

Comprehensive nursing interventions may have the potential to significantly mitigate complications associated with vascular interventional treatment, alleviate patients’ anxiety and depression and improve overall patient comfort, quality of life.

## INTRODUCTION

Vascular surgical operation represents a prevalent therapeutic strategy for addressing cardiovascular diseases and vascular lesions. With the continuous progress in medical practices and radiological technologies, interventional surgery has gained significant recognition and acceptance. Presently, a substantial proportion of vascular surgical procedures employ interventional techniques. Conditions that were previously considered inoperable or exceedingly difficult to manage can now be effectively treated through these advanced methodologies. Interventional surgery offers a minimally invasive treatment alternative for vascular disorders, yielding advantages such as diminished trauma, expedited recovery, enhanced precision in localization and the ability to address a variety of complex challenges.^1^-^3^

Nonetheless, interventional therapy is associated with inherent risks and patients may encounter various post-surgery complications, such as infection, hemorrhage and thrombosis. These complications not only increase hospitalization expenses and prolong the duration of stay but also impose a psychological strain on patients. Additionally, they may negatively influence patients’ compliance with subsequent treatments, ultimately affecting their survival outcomes and overall prognosis.^4^-^6^ The nursing plan executed in clinical practice plays a vital role in promoting patient recovery. Providing high-quality nursing care during the perioperative period assists patients in understanding information regarding their condition and the perioperative period.^7^

This understanding foster improved collaboration with medical personnel and contributes to a more effective recovery from illness.^8^ Consequently, it is essential to underscore the importance of perioperative nursing for vascular interventional therapy to mitigate complications and improve postoperative recovery outcomes for patients. The objective of this study was to assess the effects of comprehensive nursing interventions on patients undergoing vascular interventional therapy. It aimed to elucidate the tangible outcomes of comprehensive nursing on patient prognosis and to provide a scientific basis for clinical nursing decision-making.

## METHODOLOGY

This research utilizes a retrospective design, involving a selection of 80 patients who underwent interventional treatment in the vascular surgery department of The First Medical Center of Chinese PLA General Hospital from June 2022 to June 2024. The patients were categorized into two groups according to the nursing methodologies employed: a control group, which received routine perioperative nursing care and an observation group, which was provided with comprehensive nursing care, with each group consisting of 40 patients. In the control group, there were 24 males and 16 females, aged 48 to 75 years, with an average age of (59.35 ± 7.81) years; In the observation group, there were 26 males and 14 females, aged 46 to 73 years, with an average age of (58.03 ± 7.12) years. There was no statistically significant difference in the comparison of the general information of the two groups of patients (p>0.05).

### Ethical approval:

The study was approved by the Institutional Ethics Committee of The First Medical Center of Chinese PLA General Hospital (No.: S2019-283-02; Date: November 28, 2019) and written informed consent was obtained from all participants.

### Inclusion criteria:

- Patients aged 18 years or older who are scheduled for interventional operation.
- Good mental state and able to communicate normally.
- Informed consent was obtained from the patients and their families and the patients were willing to undergo surgery and regular follow-up.
- Those with complete clinical data.

### Exclusion criteria:

- Those with mental disorders who are unable to communicate normally.
- Those with malignant tumors, severe infectious diseases, or other major illnesses.

### Nursing methods:

### Nursing Interventions for the Control Group:

Routine perioperative nursing care was administered, encompassing health education for patients prior to their surgical procedures. The education included pathogenic factors, surgery-related knowledge and precautions during the perioperative period. Patients received assistance in completing the preoperative examination and their vital signs were meticulously monitored throughout the perioperative process. Any irregularities were immediately communicated to the attending physician for appropriate treatment. Additionally, patients were encouraged to engage in functional exercises, instructed to diligently follow medical recommendations concerning medication and offered general dietary advice.

### Nursing Interventions for the Observation Group:

A specialized interventional treatment nursing team was formed, led by a nurse possessing significant expertise. All the members of the team have been trained and qualified to join the team.

### Preoperative nursing:

All requisite examinations for the patient were conducted and customized health education that reflects the individual circumstances of each patient was provided. The patient was informed of the necessary precautions and was engaged to address any concerns regarding the surgery and to ensure that the patient is in optimal physical and mental health.

### Intraoperative nursing:

Intravenous access was established, thrombolytic drugs were prepared and injected and the patient’s vital signs were closely monitored, with any abnormalities promptly reported to the doctor. The catheter was properly fixed and the puncture point was dressed.

### Postoperative care:

A reasonable diet plan was implemented; Vital signs such as blood pressure, pulse and respiration were monitored. The puncture site was examined for signs of bleeding or subcutaneous hematoma and the doctor was notified of any abnormalities; The plan for rehabilitation exercises was formulated according to the patients’ actual conditions and they were encouraged to perform functional exercises; The patient was instructed to use analgesic drugs according to the doctor’s advice.

### Complication nursing:

The patient’s body temperature was closely monitored for changes and the incision was closely inspected for signs of infection. Elderly patients were encouraged to cough and pat the chest and back to prevent pulmonary complications. During the postoperative period, patients were instructed to turn over and massage to prevent the occurrence of bedsores.

### Discharge instructions:

Patients or their family members were instructed to strictly follow the doctor’s advice, to take the medication regularly and to re-examine regularly. Both patient cohorts engaged in a nursing intervention lasting one month and were subsequently observed for a duration of three months.

### Observation indicators:

### Incidence of Complications:

The occurrence of complications in both groups of patients was recorded. Prior to the intervention and at the end of the follow-up period, the General Comfort Questionnaire (GCQ) and the Health-Promoting Lifestyle Profile II (HPLP-II) were employed to assess the comfort levels and quality of life among the two patient groups. The Self-Rating Anxiety Scale (SAS) and the Self-Rating Depression Scale (SDS) were employed to compare the psychological conditions of the two patient groups both before the intervention and at the end of the follow-up period. A self-created satisfaction survey scale was used to assess patients’ contentment with the nursing care they receive. All the questionnaire was given to the participants for investigation and then collected in about five minutes.

### Statistical analysis:

Statistical analysis of the data from the two patient cohorts was performed utilizing SPSS version 21.0. The measurement data were presented as *X̄*±*S* and comparisons between the two groups were executed using an independent samples t-test. The enumeration data were represented as [n (%)] and verified using the c²test. A p-value of less than 0.05 was deemed statistically significant.

## RESULTS

The incidence of complications in the observation group was 10%, which is significantly lower than the 30% observed in the control group (p<0.05) (Table-I). The comparison of GCQ scores between the two groups before the intervention showed no statistically significant difference (p>0.05); At the end of the intervention, the GCQ score of the observation group was higher than that of the control group and the difference was statistically significant (p<0.05) (Table-II).

**Table-I:** Comparison of postoperative incidence of complications between the two groups of patients [n(%)].

Group	Hemorrhage, Hematoma	Infection	Radiation-Induced Hypotension	Thromboembolism	Limb Swelling	Incidence of Complications
Observation Group (n = 40)	1(2.50)	0(0.00)	1(2.50)	1(2.50)	1(2.50)	4(10.00)
Control Group (n = 40)	4(10.00)	1(2.50)	2(5.00)	2(5.00)	3(7.50)	12(30.00)
*χ*²						4.762
P						0.029

**Table-II:** Comparison of GCQ scores before and after intervention between the two groups of patients (*X̄*±*S*).

Item	Observation Timing	Observation Group	Control Group	t	P
Physiology	Before Intervention	10.20 ± 0.99	10.02 ± 0.92	0.585	0.560
	After Intervention	17.23 ± 1.03	15.10 ± 1.01	9.350	<0.001
Environment	Before Intervention	14.65 ± 1.10	14.40 ± 1.08	1.026	0.308
	After Intervention	25.58 ± 1.13	20.43 ± 1.26	19.259	<0.001
Psychological and Mental	Before Intervention	24.13 ± 1.42	24.40 ± 1.45	0.859	0.393
	After Intervention	34.15 ± 1.51	29.38 ± 1.48	14.278	<0.001
Socio-Cultural	Before Intervention	16.23 ± 1.62	16.43 ± 1.50	0.572	0.569
	After Intervention	29.28 ± 1.83	25.05 ± 1.72	10.642	<0.001
Total Score	Before Intervention	65.20 ± 2.84	65.30 ± 2.34	0.171	0.864
	After Intervention	106.23 ± 2.74	89.95 ± 2.80	26.267	<0.001

There was no statistically significant difference in HPLP-II scores between the two groups before the intervention (p>0.05); At the end of the intervention, the HPLP-II score of the observation group was higher than that of the control group and the difference was statistically significant (p<0.05) (Table-III).

**Table-III:** Comparison of HPLP-II Scores Before and After Intervention Between Two Groups of Patients (*X̄*±*S*).

Item	Observation Timing	Observation Group	Control Group	t	P
Health Responsibility	Before Intervention	13.90 ± 2.02	14.63 ± 1.66	1.753	0.084
	After Intervention	31.05 ± 1.30	27.60 ± 1.34	11.706	<0.001
Exercise	Before Intervention	14.83 ± 0.98	14.93 ± 0.92	0.470	0.640
	After Intervention	29.93 ± 4.37	24.13 ± 3.01	6.910	<0.001
Interpersonal Relationships	Before Intervention	15.78 ± 4.25	15.15 ± 2.93	0.766	0.446
	After Intervention	31.80 ± 4.28	28.20 ± 3.00	4.357	<0.001
Nutrition	Before Intervention	15.63 ± 3.66	15.08 ± 2.91	0.744	0.459
	After Intervention	31.53 ± 4.06	28.08 ± 2.90	4.378	<0.001
Total Score	Before Intervention	60.13 ± 7.66	59.78 ± 6.07	0.226	0.821
	After Intervention	124.30 ± 12.46	108.00 ± 8.60	6.810	<0.001

There was no statistically significant difference in SAS and SDS scores between the two groups before the intervention (p>0.05); At the end of the intervention, the SAS and SDS scores of the observation group were lower than those of the control group and the difference was statistically significant (p<0.05) (Table-IV). The satisfaction rate of patients in the observation group was 95%, which was higher than the 80% in the control group and the difference was statistically significant (p<0.05) (Table-V).

**Table-IV:** Comparison of SAS and SDS Scores Between Two Groups of Patients (*X̄*±*S*).

Item	Observation Timing	Observation Group	Control Group	t	p
SAS	Before Intervention	53.05 ± 7.00	52.86 ± 6.94	0.116	0.908
	After Intervention	43.357 ± 6.20	47.90 ± 6.07	3.319	0.001
SDS	Before Intervention	54.45 ± 5.86	54.82 ± 6.46	0.268	0.789
	After Intervention	41.63 ± 6.20	45.72 ± 5.90	3.021	0.003

**Table-V:** Comparison of Patient Satisfaction Between Two Groups [n(%)]

Group	Very Satisfied	Fairly Satisfied	Satisfied	Uncertain	Dissatisfied	Total Satisfaction Rate
Observation Group	27(67.50)	7(17.50)	4(10.00)	2(5.00)	0(0.00)	38(95.00)
Control Group	21(52.50)	7(17.50)	4(10.00)	3(7.50)	5(12.50)	32(80.00)
*χ* ^2^						4.114
*P*						0.043

## DISCUSSION

The present study evaluated the efficacy of a comprehensive nursing model in perioperative care for patients undergoing vascular interventional surgery, with findings demonstrating that this model significantly reduces postoperative complications, improves patient comfort (higher GCQ scores), enhances health-promoting behaviors (higher HPLP-II scores), alleviates negative emotions (lower SAS and SDS scores), and increases nursing satisfaction compared to conventional care (all P<0.05). These results highlight the multifaceted benefits of comprehensive nursing, which we further contextualize below through comparisons with existing literature, followed by an analysis of the study’s novelty, clinical relevance, strengths, and future directions.

Our finding that comprehensive nursing lowers complication rates aligns with the research by Bao CY et al., which reported that comprehensive perioperative care was effective in reducing complications and improving patient comfort and quality of life.^9^ A meta-analysis incorporating 18 randomized controlled trials involving a total of 1,954 patients (967 in the comprehensive nursing group and 987 in the conventional nursing group) demonstrated that patients receiving comprehensive nursing interventions had significantly lower complication rates compared to those receiving conventional care (4.21% vs 19.01%).^10^ Gou et al. applied comprehensive nursing intervention in the interventional treatment of liver cancer in cirrhosis and found that it could significantly improve the quality of life of patients and reduce the incidence of postoperative complications, which also supports the conclusions of this study.^11^

At the heart of our care model lies preoperative psychological preparation - a crucial process where nurses engage patients in personalized counseling sessions to ease their fears about surgery. The elevation in GCQ scores (indicating better physical comfort) observed in our study echoes regional research by Ren L et al., which showed that among patients undergoing ENT surgery, those who received standardized care combined with Sops had significantly higher GCQ scores than the control group.^12^ Similarly, Kaplan et al., in a randomized controlled trial, revealed that individualized care based on comfort theory had a positive impact on the comfort, satisfaction, and sleep quality of ICU patients.^13^ In another RCT, Tan JQ et al. showed that in the perioperative period of chronic suppurative otitis media, after a series of perioperative nursing measures to optimize preoperative preparation, prevent postoperative complications, reduce stress and enhance recovery, the GCQ score of the experimental group was significantly higher than that of the control group [88 (84-100) vs. 83 (78.25-92.25)].^14^

Our data showing higher HPLP-II scores (indicating better health responsibility, nutrition, and activity) align with international evidence: a systematic review by Sanson et al. concluded that health education (a core element of our model) increases patient engagement in self-care.^15^ In terms of nursing satisfaction, our results mirror those of previous studies, which found that extended nurse-patient communication increased satisfaction due to enhanced trust.^16^-^18^ However, their study focused more on communication duration, whereas ours further linked satisfaction to personalized care (e.g., tailored dietary advice for hypertensive patients). Another study has shown that patients who received comprehensive care during the perioperative period had significantly better quality of life, less pain, lower anxiety and depression, fewer adverse reactions, and higher satisfaction compared to those who received conventional care.^19^

This study addresses critical gaps in the existing literature by providing a multidimensional evaluation of patient outcomes encompassing complications, comfort levels, and health-promoting behaviors, which have traditionally been examined in isolation. Its significant contribution lies in providing tailored methods for elderly patients with comorbidities. Patients who need vascular interventional surgery are mostly middle-aged and elderly individuals, with many underlying diseases and high expectations for postoperative quality of life.^20^ Furthermore, the research elucidates the underlying mechanisms of the nursing model’s effectiveness by demonstrating how enhanced nurse competencies through specialized training and improved patient adherence through psychological support synergistically optimize outcomes. Clinically, the findings hold substantial relevance by demonstrating potential cost savings for healthcare institutions through reduced need for reinterventions. The model offers nurses a structured framework to enhance care delivery, particularly valuable in resource-constrained settings.

### Limitations:

It includes a limited sample size and a brief follow-up period. In subsequent clinical research endeavors, we intend to augment the sample size and prolong the follow-up duration to improve the thoroughness of the investigation. This approach will facilitate a more objective evaluation of the benefits and drawbacks associated with the comprehensive nursing model within the framework of vascular surgery interventional treatment, thereby ultimately serving a larger patient population.

## CONCLUSIONS

In conclusion, comprehensive nursing interventions for patients receiving vascular interventional therapy may significantly minimize surgical complications, mitigate adverse psychological conditions such as anxiety and depression and improve patients’ comfort, quality of life and overall satisfaction with their care.

### Authors’ Contributions:

**JZ:** Conceived and designed the study. Literature search,

**DC:** Collected the data and performed the analysis. Critical Review.

**XR:** Writing of the manuscript and is responsible for the integrity of the study.

All authors have read and approved the final manuscript.
